# Cases in Mercy Hospital, Surgical Wards

**Published:** 1871-10

**Authors:** 


### CLINICAL CASES IN MERCY HOSPITAL, SURGICAL WARDS.

Service of PROF. ANDREWS.

CASE I. BURNS, ETC.—The incidents of the great fire in this city bring to the surgeon's attention many cases, of two kinds in particular—burns and lacerated and contused wounds. This patient was engaged, on the night of the fire, in moving furniture, but becoming tired out, he lay down, and thinks he fell asleep. Suddenly he was surrounded by flames, and he had to rush through them to escape. His face is burned all over, and the thick beard and moustache are burned off. His hands are burned, in some places deeply into the flesh. The surface of his body was not affected extensively enough to produce marked constitutional symptoms. A woman, however, was brought into the Hospital with the entire surface of her body lightly burned, and she died without reaction from the shock. Destruction of ⅛ of the skin is usually fatal.

In the treatment it is necessary to reduce the suppuration which is proceeding on the face. For this purpose the face should be washed with the following mixture:

℞ Glycerine, } àà ℥ jv.

Water, }

Tannin, grs. xvj., vel. xxiv.

Carbolic Acid, ℈ iv.

M.

If this causes smarting, more water may be added. After being washed, the face is to be covered with simple cerate, or lard, mixed with carbolic acid, 20 grs., to the ounce of cerate.

The parts lightly burned may be covered with sweet oil or cotton soaked in oil. Sometimes, to exclude the air, the burned surface may be painted with white lead mixed with oil; lead poisoning need not be feared from a few days continuance. The dressings on cloth which have been applied to the hands cause intense pain when they are changed. To avoid this, the carbolated cerate is to be used, which softly covers the exposed nerves and does not harden or stick to the surface.

---

Case II. Operation for Removal of Necrosed Bone.—This man received a wound at Gettysburg, from a bullet, which entered just above the knee, in front, and produced a comminuted fracture of the thigh. Perhaps one-half of such patients died at once, but cases are still appearing, and are usually troublesome. Three fistulæ are established in the limb, and fragments of bone are issuing; a piece of necrosed bone can be felt in the fistula, just above the knee. Enlarging the opening by a probe and scalpel until the bone could be felt with the finger, a piece of necrosed bone having sharp angles unlike those usual in disease, was removed with the forceps. Another piece has spiculated and come off since the wound was received. No other sequestra were found, but it was not deemed advisable to sew up the fistulæ, as they would have to heal by granulation.

